# Assessing student satisfaction with university policies related to in-person classes in the era of COVID-19: a case study from Korea

**DOI:** 10.1038/s41598-025-92360-7

**Published:** 2025-03-22

**Authors:** Sungyo Jung, Yoojin Cho, Jinhyun Kwon, Yeram Yang, Jaewon Lee, Sungkyoon Kim

**Affiliations:** 1 Department of Environmental Health Sciences, Graduate School of Public Health, Seoul National University, Seoul, Republic of Korea; 2 Institute of Health and Environment, Seoul National University, Seoul, Republic of Korea

**Keywords:** COVID-19, In-person class, Satisfaction and anxiety, University education, Online survey, Multiple logistic regression analysis, Combined effect, Pandemic response in education, Health policy, Public health, Quality of life

## Abstract

This study assessed key determinants of student satisfaction with university infection prevention policies during the expansion of in-person classes amid the COVID-19 pandemic. Conducted at a South Korean university, the research surveyed 386 undergraduate and graduate students, examining the impact of health literacy, protective behavior, risk perception, institutional support, and engagement with COVID-19 information on satisfaction. Findings indicate that among online-only students, vaccination status significantly influenced satisfaction levels, suggesting that perceived safety plays a key role in mitigating pandemic-related concerns. For students attending in-person classes, frequent engagement with COVID-19 notifications and willingness to use a university-provided COVID-19 information database were strongly associated with higher satisfaction. Additionally, the study reveals a passive approach to information acquisition, highlighting students’ preference for institution-driven communication rather than independent information-seeking. These results underscore the importance of clear, structured institutional communication and proactive support systems in enhancing student satisfaction and well-being. The findings provide actionable recommendations for universities to develop strategic communication policies, expand institutional support, and improve student engagement, particularly in collectivist cultures where institutional guidance is central to decision-making.

**Supplementary Information:**

The online version contains supplementary material available at 10.1038/s41598-025-92360-7.

## Introduction

The COVID-19 pandemic significantly disrupted higher education worldwide, leading universities to rapidly adopt new strategies to ensure educational continuity while prioritizing the health and safety of students and staff^1–3^. Initially, most institutions, including those in South Korea, shifted to online learning to prevent the spread of the virus^1,4–7^. While online learning facilitated the continuation of education during the pandemic, it also posed challenges such as decreased student engagement, reduced interaction, and limitations in conducting practical, hands-on courses^1,3,7–11^.

In South Korea, universities adopted a phased approach to reintroducing in-person classes starting in September 2021, marking a significant shift from the exclusive reliance on online education^12^. This transition aimed to address the limitations of remote learning and restore the full functionality of educational and research activities. The university under study revised its COVID-19 prevention guidelines to ensure a safe return to in-person instruction while minimizing the risk of infection on campus. These revised guidelines included stringent measures such as mandatory temperature checks, regular sanitization of facilities, social distancing in classrooms, and enhanced ventilation.

The abrupt expansion of in-person classes posed a unique set of challenges for both students and educational institutions. While previous studies have explored student perceptions, such as anxiety or satisfaction with online learning during the pandemic, there is a lack of comprehensive research on how students perceive the transition to in-person classes, particularly in terms of the effectiveness of infection prevention measures^13–15^. This study addresses this gap by evaluating student satisfaction with university policies related to in-person classes during the COVID-19 pandemic. It provides insights into how students reacted to the return of physical classes and how institutional guidelines influenced their overall satisfaction and anxiety levels.

Globally, educational institutions have faced immense challenges in balancing the need for continued education with the imperative to protect public health^2,10^. The findings of this study contribute to the broader discourse on effective educational strategies during pandemics, offering valuable data that can inform future policy-making in higher education.

### Key differences between online and in-person classes

A primary distinction in the university’s infection prevention guidelines between online and in-person classes was the mode of attendance. During the period of online classes, students engaged in coursework remotely without needing to visit campus. University guidelines focused solely on general COVID-19 prevention measures, such as infection response protocols and preventive measures for students living off-campus.

In contrast, the reintroduction of in-person classes required students to physically attend classes on campus, following comprehensive safety protocols. These measures included mandatory mask-wearing, social distancing, temperature checks at university facilities, daily health monitoring through mobile application check-ins, and tracking individual movement on campus. These contrasting approaches created unique learning environments: while the online model minimized physical presence, the in-person model emphasized a carefully controlled, safe return to regular academic operations. Understanding these policy distinctions and their impact on student satisfaction is essential for assessing the effectiveness of institutional responses during health crises.

### Research questions

To guide this study, the following research questions were formulated:

1. What are the primary factors influencing student satisfaction with university COVID-19 guidelines during the transition from online to in-person classes?
2. What are the specific concerns and anxieties students experience regarding in-person class attendance, and how do these factors affect their overall satisfaction with the university’s pandemic response?
3. How does student engagement with university-provided COVID-19 information impact their satisfaction and perceived safety during in-person classes?
4. What strategies can educational institutions implement to enhance student satisfaction and reduce anxiety during the transition to in-person learning amidst ongoing pandemic conditions?

This study addresses these questions by identifying factors that can enhance student satisfaction in the context of the university’s in-person class operations and suggests actionable insights to improve satisfaction and reduce anxiety. Through examining these determinants, the research aims to provide valuable recommendations that support universities in fostering a safer, more satisfying learning environment during transitions from online to in-person classes amidst pandemics such as COVID-19.

## Review of relevant literature

This study builds upon a systematic review and analysis of existing research assessing students’ perceptions, such as anxiety or satisfaction, to institutional interventions like infection prevention policies or institutional support in educational environments during the COVID-19 pandemic. By synthesizing findings from prior studies, this section establishes the theoretical foundation for identifying key determinants of student satisfaction and informs the design of the survey instrument and data interpretation process used in the present research.

### Systematic review process

A systematic search for relevant studies was conducted using databases such as PubMed, Scopus, Web of Science, and Google Scholar. The search criteria included:

1. Publication date: Studies published between January 2020 and September 2021.
2. Study design: Quantitative, qualitative, or mixed-method research.
3. Sample size: Studies with a minimum of 100 participants.
4. Language: Articles published in English.

A total of 167 studies were retrieved, of which 9 high-quality studies were selected for analysis based on relevance to the topic, methodological rigor, and availability of detailed findings. Screening criteria ensured the inclusion of research examining student satisfaction with infection prevention measures in educational settings, such as communication efficiency, perceived safety, and trust in institutional guidelines.

After conducting the online survey for this study, the findings were compared to prior research to identify similarities. Additionally, to interpret the results, three relevant studies published after the survey period were reviewed. Thus, a total of 12 studies—nine used for survey design and three for result interpretation—were analyzed. A summary of these studies is provided in Table 1.

**Table 1:** Summary of studies on student perceptions to institutional policies and support during the COVID-19 pandemic.

Study	Country	Sample	Sample Size	Learning mode	Key focus	Method	Findings
Aguilera-Hermida (2020)	USA	College students	270	Online	Emergency online learning and satisfaction	Online survey	Emphasizes communication clarity and trust in guidelines
Ives (2021)	USA	University students	320	Online	Remote instruction experience & policy satisfaction	Online survey	Highlights the importance of clear and consistent policies
Islam et al. (2020)	Bangladesh	University students	431	Online	Psychological response to pandemic measures	Online survey	Perceived safety and mental health impact were critical
Williams & Corwith (2021)	USA	Teachers, students, parents (Middle & high school)	180	Hybrid	Online learning efficacy & community-building	Qualitative analysis	Engagement and safety perception influence satisfaction
Birhanu et al. (2021)	Ethiopia	University students	512	Online	Risk perception & attitudes towards COVID-19	Online survey	Trust in guidelines improves compliance and satisfaction
Maqableh & Alia (2021)	Jordan	University students	640	Online	Online learning experience and satisfaction	Online survey	Technical difficulties and lack of engagement reduced satisfaction
Alzahrani & Seth (2021)	UK	University students	245	Online	LMS usage satisfaction	Online survey	Self-efficacy and service quality influenced satisfaction
Tang et al. (2021)	China	School students	584	Online	Mental health & communication during school closures	Online survey	Parent–child discussions and safety perceptions were critical
Rattay et al. (2021)	Germany	University students	690	Online	Risk perception & protective behavior	Online survey	Higher awareness improved protective behaviors and satisfaction
Punjani & Mahadevan (2022)	India	University students	275	Online	Awareness of COVID-19 & self-efficacy	Online survey	Awareness and self-efficacy correlated with higher satisfaction
Tabatabaeichehr et al. (2022)	Global	Medical students	None	Online	E-learning satisfaction	Systematic review	Instructor involvement and interactive content improved satisfaction
Conrad et al. (2022)	Canada	University students	410	Online	Perceptions of online learning difficulty	Survey & interviews	Students with technical barriers reported lower satisfaction

### Thematic analysis and key findings

The selected studies underwent thematic analysis to identify recurrent themes and influencing factors. Special attention was paid to heterogeneity among studies, which was addressed through subgroup and sensitivity analyses to ensure robustness.

#### Key determinants of student perceptions

Institutional responses to COVID-19 have significantly influenced student satisfaction and compliance with university guidelines. Key factors shaping student perceptions include communication efficiency, perceived safety, trust in institutional guidelines, institutional support, health literacy, and psychological well-being.

##### Communication efficiency

Aguilera Hermida (2020) and Ives (2021) emphasized the importance of clear, consistent communication and trust in institutional guidelines for maintaining student satisfaction^1,3^. Alzahrani & Seth (2021) also noted that service quality and consistency of institutional updates are key drivers of student satisfaction in hybrid learning environments^13^.

##### Perceived safety

Studies by Islam et al. (2020) and Tabatabaeichehr et al. (2022) highlighted the critical role of perceived safety measures, such as mask mandates, ventilation, and social distancing, in enhancing students’ confidence in attending classes^4,14^. Their findings align with broader research emphasizing the psychological importance of a safe learning environment.

##### Trust in institutional guidelines

Birhanu et al. (2021) demonstrated that students with higher trust in guidelines exhibited greater compliance and satisfaction^7^. Williams & Corwith (2021) focused on institutional transparency and its role in fostering trust in policies^5^.

##### Institutional support

Rattay et al. (2021) highlighted the importance of mental health resources, flexible attendance policies, and accessible learning materials^16^. Punjani and Mahadevan (2022) found that community engagement and institutional flexibility significantly impacted student satisfaction^6^.

##### Health literacy and risk perception

Tang et al. (2021) and Conrad et al. (2022) noted that higher health literacy reduced anxiety and enhanced compliance with safety protocols^15,17^. Rattay et al. (2021) further confirmed that knowledge of COVID-19 risks improved protective behaviors and adherence to institutional policies^16^.

##### Psychological well-being and engagement barriers

Maqableh & Alia (2021) and Conrad et al. (2022) documented how technical challenges and limited interaction during online learning led to reduced satisfaction^8,15^. Tang et al. (2021) highlighted the role of parent-student discussions in mitigating anxiety related to pandemic-induced school closures^17^.

#### Theoretical framework and research design integration

Findings from previous studies confirm that the six key factors identified above significantly influence students’ perceptions to university policies. Through the literature review, this study concludes that student satisfaction with university policies is primarily determined by perceived safety and trust in institutional guidelines. These perceptions, in turn, are shaped by communication efficiency and students’ health literacy and risk perception. Additionally, institutional support serves as a moderating factor, either reinforcing or weakening these relationships depending on the quality and extent of university-led engagement.

Accordingly, these insights were systematically incorporated into the research design to develop the online survey measures and questionnaire structure. In this study, students’ satisfaction with university policies is evaluated within the framework of perceived safety and trust in institutional guidelines, as these constructs were determined to encapsulate satisfaction with institutional COVID-19 measures. Thus, the survey includes only questions assessing students’ perceptions of university guidelines under these measure variables. Furthermore, the survey is structured to analyze the influence of contributing factors and quantify their impact on students’ perceptions of university-led COVID-19 policies.

By integrating these theoretical constructs into the survey framework, this study ensures a comprehensive assessment of the determinants of student satisfaction, thereby contributing to future policy improvements for managing in-person learning environments during public health crises.

## Methods

### Sample

The study was conducted at a large university in South Korea, known for its diverse student population and wide range of academic disciplines. As of 2021, the university had approximately 27,000 enrolled students, including both undergraduate and graduate students across various faculties. The institution offers numerous degree programs in fields such as natural sciences, social sciences, humanities, and engineering, providing a broad academic context for this study.

This study targeted undergraduate and graduate students currently enrolled at the university during the first semester of in-person classes following the COVID-19 pandemic. The sample was recruited through an online survey administered in September 2021, following the university’s implementation of revised COVID-19 guidelines to support the transition from online to in-person classes. A total of 386 responses were collected, exceeding the minimum required sample size of 199 determined using the G*Power 3.1.9.7 program for regression analysis, with a medium effect size (0.15), a significance level of 0.05, power of 0.95, and a maximum of 15 predictors^18^.

In accordance with Institutional Review Board (IRB) regulations, this study ensured participant anonymity and minimized the collection of personal data. As a result, specific demographic variables such as age, gender, and nationality were not directly collected. However, degree level (undergraduate or graduate) and faculty affiliation were included to allow for demographic-based adjustments during analysis. Degree level provides a proxy for age estimation, as most undergraduates are between 18–24 years old, while graduate students typically range from mid-20 s to early 30 s.

To provide additional context, institutional statistics indicate that the university’s student body consists of approximately 55% male and 45% female students. The international student population includes students from 112 countries, accounting for 1.08% of undergraduates and 7.97% of graduate students, resulting in an overall international student enrollment of 4.09%. While these institutional data provide a broader demographic overview, the absence of individually collected gender and nationality data is acknowledged as a study limitation.

### Measures and questionnaire

This study assessed key variables influencing student satisfaction and engagement with university infection prevention policies during the expansion of in-person learning. The survey design was informed by WHO guidelines and previous research, ensuring alignment with established public health frameworks^19^.

#### Measures

The survey included ten core variables, derived from the WHO’s Survey Tool and Guidance for COVID-19 and insights from previous studies^19^. The measures and corresponding survey items were structured as follows:

1. Preventive Behaviors (Q2, Q3, Q4): Assessed students’ vaccination status, participation in university-administered COVID-19 testing, and engagement with university-provided COVID-19 updates.
2. Policy Awareness (Q6): Evaluated students’ awareness of COVID-19 policies implemented by the university.
3. Affect (Q7): Assessed the impact of the expansion of in-person learning by measuring the actual number of students attending in-person classes.
4. Perceived Safety & Trust in Institutions (Q8, Q8-1): Examined students’ confidence in the university’s infection prevention policies and satisfaction with institutional guidelines.
5. Risk Perception (Q9, Q10): Investigated students’ concerns about COVID-19 exposure risks in campus facilities and shared spaces.
6. Health Literacy (Q11, Q12-1, Q13, Q14): Explored students’ information-seeking behaviors, frequency of acquiring COVID-19-related knowledge, reasons for not actively searching for information, and demand for additional information.
7. Communication Effectiveness (Q4, Q5, Q6, Q6-1): Evaluated students’ perceptions of the clarity and accessibility of COVID-19-related information, such as infection updates and prevention policies, provided by the university.
8. Institutional Support (Q3, Q4, Q14, Q15): Assessed students’ engagement with university-provided resources, expectations for further support, and specific demands regarding COVID-19-related information.

Each measure was assessed using Likert-scale, binary (Yes/No), or multiple-choice responses, depending on the nature of the variable. A detailed breakdown of survey items is provided in Table 2.

**Table 2:** Variables used to assess student perceptions and behaviors during the expansion of in-person classes amid the COVID-19 pandemic.

Measures	Survey items	Related questions
Demographics	Degree course	Q1
	Faculty affiliation	Q1-1
Preventive behaviors	Vaccination status	Q2
	On-campus COVID-19 testing experience	Q3
	Monitoring COVID-19 updates on campus	Q4
Policy awareness	Awareness of university COVID-19 guidelines	Q6
Affect	Participation in in-person classes	Q7
Perceived safety/trust in institutions	Confidence in university infection prevention policies	Q8
		Q8-1
Risk perception	Concerns about infection risks in facilities	Q9
		Q10
Health literacy	Searching experience for COVID-19 information	Q11
		Q12-1
		Q13
	Hoping to use COVID-19 database	Q14
Information sources	Sources of information on policy changes, infection prevention guidelines, and COVID-19	Q5
		Q6-1
		Q12
Communication effectiveness	Perceived effectiveness of institutional communication	Q4
		Q5
		Q6
		Q6-1
Institutional support	Institutional support effectiveness in infection prevention and student anxiety mitigation Student demand for additional institutional support	Q3
		Q4
		Q14
		Q15

#### Questionnaire

The survey questionnaire consisted of 18 items, structured to evaluate key determinants of student satisfaction with university COVID-19 policies. The items were developed based on previous research and WHO guidelines to ensure validity and relevance.

The questionnaire was organized into four primary domains:

1. Demographics: Collected basic background information, including degree level and faculty affiliation.
2. Policy Perceptions & Risk Awareness: Assessed students’ knowledge of COVID-19 guidelines, perceived safety, and trust in institutional policies.
3. Health Literacy & Information-Seeking Behavior: Measured students’ engagement with COVID-19-related resources, patterns of information-seeking, and willingness to use university-provided information.
4. Institutional Support & Communication Effectiveness: Examined student participation in university-led COVID-19 initiatives, expectations for institutional support, and the effectiveness of university communication strategies.

The survey was administered online via Google Forms from September 17 to 23, 2021, following the university’s expansion of in-person learning. A total of 386 responses were collected, ensuring a sufficiently large sample size for statistical analysis. The structure of the survey and corresponding variable classifications are detailed in Tables 2 and 3. Further details on response distributions are provided in Table S1.

**Table 3:** Reclassified results of survey data for multiple logistic regression analysis (N = 386).

Question	Category	Answer	Response (%)	p-value^a^
Q1	Degree course	Undergraduate	285 (73.83)	0.25
		Graduate	101 (26.17)	
Q2	Vaccination status	No	118 (30.57)	< 0.01
		1^st^	138 (35.75)	
		2^nd^	130 (33.68)	
Q3	On-campus COVID-19 testing experience	No	87 (22.54)	0.20
		Yes	299 (77.46)	
Q4	Monitoring COVID-19 updates on campus	Never	35 (9.07)	< 0.01
		Seldom	136 (35.23)	
		Often/every time	215 (55.70)	
Q6	Awareness of university COVID-19 guidelines	Never checked	143 (37.05)	0.64
		Know some	215 (55.70)	
		Know everything	28 (7.25)	
Q7	Participation in in-person classes	No	121 (31.35)	< 0.01
		Yes	265 (68.65)	
Q8	Satisfaction with overall university guidelines	Unsatisfied	73 (18.91)	-
		Neutral	235 (60.88)	
		Satisfied	78 (20.21)	
Q11	Searching experience for	No	279 (72.28)	0.08
	COVID-19 information	Yes	107 (27.72)	
Q14	Hoping to use COVID-19 information	No	104 (26.94)	< 0.01
	database provided by the university	Yes	282 (73.06)	

a: The p-value was estimated using the Chi-square test to assess the independence between satisfaction with the guidelines and each factor.

### Study procedure

#### Process of study

An online survey, available from September 17th to 23rd, 2021, received 386 voluntary responses from current students. This survey followed 18 months of exclusive remote instruction initiated by the WHO’s pandemic declaration and three months post the announcement of expanded in-person instruction, being conducted two weeks after its implementation. The survey, created using Google Forms, was disseminated to students via community notices and campus-wide emails.

#### Ethical consideration

The study’s data collection and survey methods were approved by the IRB of Seoul National University (IRB No. 2107/003–007). Informed consent obtained from all participants. Participation commenced only after students acknowledged understanding and consented to partake in the study, with the survey page closing automatically for non-consenting individuals. All procedures and methods employed in this study were conducted in strict accordance with the relevant guidelines and regulations set forth by the approving institution.

### Statistical analysis

#### Multiple logistic regression analysis

A multiple logistic regression analysis was conducted to evaluate the association between student satisfaction with university policies and various factors such as communication effectiveness, perceived safety, and trust in institutional guidelines^20,21^. Responses were scored using a 5-point Likert scale, where 1 indicated the lowest level of satisfaction (“very dissatisfied”) and 5 indicated the highest level of satisfaction (“very satisfied”). These responses were aggregated to generate overall satisfaction scores.

The dependent variable—satisfaction with university COVID-19 guidelines—was categorized into three groups: “unsatisfied” (very dissatisfied or dissatisfied), "neutral," and “satisfied” (satisfied or very satisfied). Independent variables included vaccination status, participation in on-campus COVID-19 testing, engagement with COVID-19-related university communications, awareness of guideline changes, attendance at in-person classes, and the use of COVID-19 literature databases. Chi-square tests were used to assess the independence of each variable in relation to student satisfaction. Details of data reclassification and Chi-square test results are presented in Table 3.

Given the partial return to in-person classes, class attendance was considered a key factor in evaluating satisfaction with COVID-19 measures. Therefore, responses were further categorized based on attendance of in-person classes to explore its moderating effect on satisfaction in the multiple logistic regression analysis. The regression analysis was conducted in three models to evaluate the stepwise effects of variables on satisfaction. Model I assessed the crude odds ratio (OR) for each variable. Model II adjusted for demographic characteristics, including degree course and department of study. Model III provided comprehensive adjustments for all variables, offering a holistic view of their impact on satisfaction. This structured approach allowed for a detailed understanding of the factors influencing student satisfaction with the university’s COVID-19 response. Analyses were performed using R software (version 4.1.2), with a p-value of less than 0.05 considered statistically significant. Confidence intervals (CIs) were calculated at the 95% level to assess the precision of the odds ratios.

#### Estimation of combined-effects by factors

To assess the influence of in-person class attendance on student satisfaction, the relative excess risk due to interaction (RERI) and ratios of odds ratios (RORs) were calculated. This approach evaluated the combined effect of class attendance with other factors, such as vaccination status, attention to COVID-19 notifications, and willingness to use COVID-19 databases. These variables were identified through logistic regression as having a significant impact on satisfaction related to class attendance. For a more detailed analysis, the responses of these variables were categorized into two-point Likert scales, as shown in Table S2. The dependent variable—satisfaction with university COVID-19 guidelines—was categorized into two groups: “unsatisfied” (very dissatisfied or dissatisfied) and "neutral or satisfied" (neutral, satisfied, or very satisfied).

We then calculated the RERI and RORs to estimate interactions on additive and multiplicative scales, respectively. This analysis was conducted using R software (version 4.1.2) and the ‘interactionR’ package, following Eq. 1 as referenced in previous studies^22–24^. A RERI value greater than 0 indicates a supra-additive interaction, while a RORs value greater than 1 suggests an intensified combined effect compared to the individual effects of the exposures.

Equation (1): Formula for calculating relative excess risk due to interaction (RERI) and ratios of odds ratios (RORs).

1 $$\begin{aligned} {\text{RERI }} = & {\text{ OR}}_{{{\text{combined exposure to all online class and positive response of factor}}}} \\ \quad & {-}{\text{ OR}}_{{{\text{exposure to only all online class}}}} \\ \quad & {-}{\text{ OR}}_{{{\text{exposure to only positive response of factor}}}} + {\text{ 1}}. \\ {\text{RORs }} = & {\text{ }}\frac{{{\text{OR}}_{{{\text{combined exposure to all online class and positive response of factor}}}} }}{{\left( {{\text{OR}}_{{{\text{exposure to only all online class}}}} \times {\text{ OR}}_{{{\text{exposure to only positive response of factor}}}} } \right)}}. \\ \end{aligned}$$

## Results

### Survey response

Among the 386 respondents, 73.83% (n = 285) were undergraduates, while 26.17% (n = 101) were graduate students. Regarding COVID-19 vaccination, 69.43% (n = 268) had received at least one dose, with 33.68% (n = 130) being fully vaccinated. Participation in on-campus COVID-19 testing was high (77.46%, n = 299). Additionally, 55.69% (n = 215) monitored campus updates on COVID-19 infection cases at least twice weekly, whereas 37.05% (n = 143) did not check university guideline updates at all.

Class attendance data revealed that 68.65% (n = 265) had attended at least one in-person class. Satisfaction with university COVID-19 guidelines varied: 20.21% (n = 78) expressed satisfaction, while 18.91% (n = 73) reported dissatisfaction. Regarding information-seeking behavior, 27.72% (n = 107) actively searched for COVID-19 information, and 73.06% (n = 282) expressed interest in a university-managed COVID-19 information database.

As the survey collected degree level and faculty affiliation but did not include specific demographic variables such as age, gender, and nationality, general university-wide demographic characteristics provide contextual reference. Institutional data indicate that the student body is approximately 55% male and 45% female, and the total international student population accounts for 4.09% of enrolled students, with a higher proportion at the graduate level.

A detailed breakdown of survey responses is provided in Table S1, outlining student engagement with university resources, adherence to safety measures, and perceived institutional support. These findings offer insight into student compliance with pandemic-related university policies, satisfaction with institutional support, and demand for additional informational resources.

### Qualitative insights into students’ COVID-19 safety concerns

Students expressed concerns regarding COVID-19 safety on campus, particularly in relation to infection risks in shared spaces, adherence to safety measures, and access to reliable information. Open-ended survey responses provided insights into perceived high-risk locations, facility-specific safety concerns, and the demand for more comprehensive COVID-19-related information from the university.

Lecture rooms and laboratories (19.42%, n = 209) were frequently identified as high-risk areas due to prolonged exposure in enclosed settings. Convenience facilities such as restaurants and cafés (31.97%, n = 344) were also commonly mentioned, with students citing poor ventilation and challenges in maintaining physical distancing. Dormitories (13.01%, n = 140) were perceived as a potential risk due to shared living spaces, while shuttle buses (14.59%, n = 157) were noted for overcrowding and limited air circulation. Additionally, indoor and outdoor sports facilities (10.04%, n = 108) raised concerns regarding mask adherence and hygiene practices for shared equipment.

Beyond location-based risks, students highlighted specific concerns about safety measures in campus facilities. Physical distancing limitations (22.19%, n = 211) were frequently mentioned, as students felt that seating arrangements and narrow hallways made distancing difficult. Mask compliance (26.39%, n = 251) was another common concern, particularly in lecture halls and dining areas where regulations were inconsistently enforced. Ventilation and air circulation (17.67%, n = 168) were perceived as inadequate, contributing to concerns about airborne transmission. Additionally, hand sanitization and disinfection (18.61%, n = 177) were cited as areas needing improvement, with students requesting more frequent cleaning of high-contact surfaces and increased availability of hand sanitizers across campus.

Regarding access to information, many students expressed a preference for centralized, transparent, and easily accessible COVID-19 updates. The most requested topics included COVID-19 vaccine efficacy and side effects (22.62%, n = 245), with students seeking up-to-date scientific findings and booster dose recommendations. Information on transmission routes and prevention methods (19.02%, n = 206) was also highly requested, as students wanted clear guidelines on minimizing personal risk.

Overall, these findings underscore the importance of institutional communication, enforcement of safety protocols, and accessibility of COVID-19-related information in shaping student perceptions of safety on campus. Addressing these concerns through improved ventilation, stricter mask enforcement, enhanced communication, and the development of a university-managed COVID-19 information platform could enhance student confidence and adherence to safety measures during in-person learning.

### Estimation of factors affecting students’ satisfaction

Table 3 presents key variables evaluated in the analysis, including vaccination status, attention to COVID-19 notifications, in-person class attendance, and willingness to use a COVID-19 database. Chi-square tests revealed a significant association between these variables and student satisfaction with university guidelines (p-value < 0.01). Three logistic regression models were applied: Model I for crude odds ratios (ORs), Model II adjusted for demographic characteristics, and Model III incorporating all variables. Comprehensive results, including adjusted odds ratios (aORs) and confidence intervals (CIs), are detailed in Table 4. An expanded analysis in Table S3 further assesses factors affecting student satisfaction, integrating in-person class attendance as an independent variable alongside those listed in Table 3.

**Table 4:** Multiple logistic regression analysis results according to the in-person class attendance.

	Model I						Model II						Model III					
	Taking class			Not taking class			Taking class			Not taking class			Taking class			Not taking class		
	OR	95% CI	P-value	OR	95% CI	P-value	aOR*	95% CI	P-value	aOR*	95% CI	P-value	aOR*	95% CI	P-value	aOR*	95% CI	P-value
Vaccination status																		
Unsatisfied	Rf			Rf			Rf			Rf			Rf			Rf		
Neutral	1.14	0.73–1.77	0.57	1.57	0.68–3.61	0.29	1.14	0.73–1.78	0.56	1.49	0.64–3.49	0.36	1.14	0.71–1.81	0.59	1.45	0.59–3.55	0.41
Satisfied	1.48	0.87–2.52	0.15	3.87	1.24–12.1	** < 0.05**	1.49	0.87–2.54	0.14	3.40	1.07–10.8	** < 0.05**	1.47	0.84–2.60	0.18	3.59	1.06–12.1	** < 0.05**
Experience of taking on-campus COVID-19 test																		
Unsatisfied	Rf			Rf			Rf			Rf			Rf			Rf		
Neutral	1.38	0.64–2.94	0.41	1.26	0.26–6.23	0.77	1.39	0.65–2.97	0.39	1.23	0.25–6.09	0.80	1.26	0.56–2.83	0.57	1.23	0.23–6.62	0.81
Satisfied	1.86	0.79–4.38	0.15	2.29	0.37–14.3	0.38	1.90	0.80–4.46	0.14	2.13	0.33–13.6	0.43	1.56	0.63–3.89	0.34	1.58	0.22–11.3	0.65
Checking message notifying information about COVID-19 infection cases on campus																		
Unsatisfied	Rf			Rf			Rf			Rf			Rf			Rf		
Neutral	2.16	1.38–3.38	** < 0.01**	0.56	0.19–1.62	0.28	2.15	1.37–3.39	** < 0.01**	0.51	0.18–1.51	0.22	1.95	1.20–3.17	** < 0.01**	0.48	0.15–1.51	0.21
Satisfied	3.22	1.80–5.78	** < 0.01**	1.33	0.32–5.59	0.70	3.20	1.77–5.77	** < 0.01**	1.13	0.26–4.87	0.87	2.81	1.52–5.23	**0.01**	1.18	0.24–5.74	0.83
Perceived information regarding university guidelines																		
Unsatisfied	Rf			Rf			Rf			Rf			Rf			Rf		
Neutral	0.87	0.52–1.45	0.60	0.50	0.19–1.31	0.16	0.85	0.51–1.43	0.54	0.49	0.18–1.32	0.16	0.73	0.42–1.27	0.27	0.53	0.19–1.48	0.22
Satisfied	0.88	0.48–1.61	0.67	0.35	0.10–1.20	0.10	0.84	0.46–1.56	0.59	0.36	0.10–1.25	0.11	0.64	0.33–1.25	0.19	0.27	0.06–1.12	0.07
Searching experience for COVID-19 information																		
Unsatisfied	Rf			Rf			Rf			Rf			Rf			Rf		
Neutral	0.75	0.38–1.47	0.40	1.06	0.27–4.21	0.93	0.74	0.38–1.45	0.38	1.02	0.25–4.05	0.98	0.58	0.28–1.21	0.15	0.83	0.19–3.72	0.81
Satisfied	1.32	0.62–2.81	0.48	2.33	0.47–11.7	0.30	1.29	0.60–2.77	0.51	2.08	0.41–10.7	0.38	0.96	0.41–2.24	0.92	2.03	0.31–13.3	0.46
Hoping to use COVID-19 database provided by the university																		
Unsatisfied	Rf			Rf			Rf			Rf			Rf			Rf		
Neutral	3.55	1.86–6.79	** < 0.01**	1.65	0.49–5.52	0.42	3.53	1.85–6.75	** < 0.01**	1.59	0.47–5.37	0.45	3.52	1.76–7.04	** < 0.01**	2.55	0.63–10.3	0.19
Satisfied	4.25	1.86–9.71	** < 0.01**	2.03	0.42–9.89	0.38	4.21	1.84–9.62	** < 0.01**	1.84	0.37–9.17	0.45	3.65	1.51–8.87	** < 0.01**	1.68	0.25–11.1	0.59

Model 1: univariate analysis of each independent variable.

Model 2: Model 1 adjusted by demographic characteristic.

Model 3: multivariable model incorporating all independent variables.

*aOR: adjusted odds ratio; Rf: reference; Class: in-person class; CI: confidence interval.

Analysis results that showed statistical significance at p-value < 0.05 are presented in bold.

The regression analysis (Table 4) found that among students attending only online classes, satisfaction significantly increased with vaccination status across all models. Model III showed an aOR of 3.59 (95% CI: 1.06–12.1, p-value < 0.05) when satisfaction shifted from “unsatisfied” to “satisfied.” In contrast, students attending in-person classes reported significantly higher satisfaction with more frequent monitoring of COVID-19 infection cases updates, yielding an aOR of 1.95 (95% CI: 1.20–3.17, p-value < 0.01) for the shift from “unsatisfied” to “neutral” and an aOR of 2.81 (95% CI: 1.52–5.23, p-value = 0.01) for “unsatisfied” to “satisfied” in Model III. Students attending in-person classes also reported higher satisfaction if they expressed willingness to use a COVID-19 database, with an aOR of 3.52 (95% CI: 1.76–7.04, p-value < 0.01) from “unsatisfied” to “neutral” and an aOR of 3.65 (95% CI: 1.51–8.87, p-value < 0.01) from “unsatisfied” to “satisfied” in Model III. Interestingly, perception of university guidelines and previous experience seeking COVID-19 information did not display statistically significant associations with satisfaction levels.

In the analysis shown in Table S3, where in-person attendance status was treated as an independent variable, online-only students exhibited a statistically significant satisfaction increase compared to those attending more than one in-person class. Model III showed an aOR of 2.83 (95% CI: 1.43–5.61, p-value < 0.01) for satisfaction change from “unsatisfied” to “neutral.” Consistent with Table 4, vaccination status significantly affected satisfaction, with an aOR of 1.83 (95% CI: 1.10–3.03, p-value < 0.05) when moving from “unsatisfied” to “satisfied.” Additionally, students monitoring COVID-19 infection case updates and those hoped to use COVID-19 information database showed significantly higher satisfaction (p-value < 0.05). Perception of university guidelines, however, was associated with lower satisfaction but lacked statistical strength, except for a single case from “unsatisfied” to “satisfied” in Model III, with an aOR of 0.53 (95% CI: 0.29–0.95, p-value < 0.05).

### Estimation of combined-effects by factors

The study identified significant differences in the impact of each factor on student satisfaction depending on in-person class attendance. To quantify the combined effect of key factors—vaccination status, frequency of checking COVID-19 notifications, and willingness to use a university-provided COVID-19 database—interaction terms were calculated. Using RERI and RORs based on Model III (Table 4), interaction effects on both additive and multiplicative scales were derived, as detailed in Table 5.

**Table 5:** Influence of in-person class attendance on satisfaction variables: vaccination status, information engagement, and database use preferences.

	Taking in-person class	Not taking in-person class	OR for not taking class versus taking class within strata of variables
Vaccination status: No	Reference	1.57 (0.44–5.58): 0.49	1.57 (0.44–5.58): 0.49
Vaccination status: Yes (1st or 2nd)	2.12 (1.00–4.50): 0.05	6.08 (2.24–16.55): < 0.01	2.88 (1.26–6.53): < 0.05
OR for Yes (1st or 2nd) versus No within taking in-person class	2.12 (1.00–4.50): 0.05	3.88 (1.05–14.39): < 0.05	
Measure of interaction on additive scale: RERI	3.39 (-1.75–8.54): 0.10		
Measure of interaction on multiplicative scale: RORs	1.83 (0.40–8.31): 0.43		
Checking information about COVID-19 cases on campus: never & seldom	Reference	8.09 (2.27–28.89): < 0.01	8.09 (2.27–28.89): < 0.01
Checking information about COVID-19 cases on campus: often & everytime	2.58 (1.34–4.95): < 0.01	2.61 (1.16–5.86): < 0.05	1.01 (0.43–2.39): 0.98
OR for often & everytime versus never & seldom within taking class	2.58 (1.34–4.95): < 0.01	0.32 (0.08–1.32): 0.11	
Measure of interaction on additive scale: RERI	-7.06 (-17.59–3.46): 0.91		
Measure of interaction on multiplicative scale: RORs	**0.12 (0.03–0.58): < 0.01**		
Hoping to use COVID-19 database provided by the university: No	Reference	4.00 (1.33–12.01): < 0.05	4.00 (1.33–12.01): < 0.05
Hoping to use COVID-19 database provided by the university: Yes	3.97 (2.06–7.65): < 0.01	6.78 (2.70–17.01): < 0.01	1.71 (0.73–3.98): 0.22
OR for Yes versus No within taking class	3.97 (2.06–7.65): < 0.01	1.69 (0.48–5.92): 0.42	
Measure of interaction on additive scale: RERI	-0.20 (-6.86–6.47): 0.52		
Measure of interaction on multiplicative scale: RORs	0.43 (0.11–1.70): 0.23		

*OR: odds ratio; RERI: relative excess risk due to interaction; RORs: ratios of odds ratios.

Analysis results that showed statistical significance at p-value < 0.05 are presented in bold.

Among the variables analyzed, none demonstrated statistically significant interactions across both scales (p-value < 0.05). However, an additive positive interaction was observed in students who solely attended online classes, where a higher vaccination status was associated with a RERI of 3.39 (95% CI: -1.75 to 8.54, p-value = 0.10). This indicates that vaccination had a moderately increased effect on satisfaction in this group, though the result did not reach statistical significance. Furthermore, frequency of checking updates on COVID-19 cases demonstrated a notable negative interaction on the multiplicative scale for in-person attendees, with a ROR of 0.12 (95% CI: 0.03 to 0.58, p-value < 0.01), suggesting that satisfaction was lower among students who frequently monitored updates. These findings reveal that while vaccination status and information engagement had combined effects on satisfaction, the direction and significance of these effects differed between students attending in-person versus online classes.

## Discussion

This study examined key determinants of student satisfaction with university COVID-19 prevention policies during the expansion of in-person classes. Consistent with prior research, critical factors included communication efficiency, perceived safety, trust in institutions, institutional support, health literacy, and risk perception. Students who actively engaged with university-provided COVID-19 information reported significantly higher satisfaction levels, highlighting the role of effective institutional communication and support in enhancing health literacy, which in turn strengthens perceived safety and trust in institutional guidelines. Additionally, a passive approach to information acquisition was observed as a unique cultural aspect in this study, likely influenced by collectivist cultural norms in Korean society, where students rely more on institutional sources rather than independently seeking information^25^.

Despite the resumption of in-person classes, engagement with preventive measures remained inconsistent. While 68.7% attended at least one in-person class, 33.2% had not reviewed the university’s infection prevention guidelines, and 30.6% were unvaccinated. However, even students with lower engagement levels in COVID-19 information and guidelines expressed a strong preference for institutionally provided COVID-19 updates (90.9%) and showed willingness to use a COVID-19 information database (73.1%), indicating reliance on institution-driven communication.

Student satisfaction with university guidelines was generally low, with 47.7% reporting dissatisfaction, 32.1% responding neutrally, and only 20.2% expressing satisfaction. Among students who had not checked institutional guidelines, 44.8% reported dissatisfaction, while 34.3% responded neutrally, suggesting that prior awareness of guidelines did not significantly impact satisfaction levels.

Findings from regression models (Table 4) indicate that for students primarily attending online classes, higher vaccination status was significantly associated with increased satisfaction levels (aOR = 3.59, 95% CI = 1.06–12.1, p-value < 0.05). This suggests that perceived health security can alleviate pandemic-related anxiety, particularly for students engaged in online learning. Similar trends have been observed in previous research emphasizing the relationship between perceived safety and student satisfaction in online learning environments^4,5,17^.

For students attending in-person classes, satisfaction significantly increased with more frequent engagement with campus COVID-19 notifications and preference for a university-provided COVID-19 database. Model 3 showed that for these students, the aOR showed 2.81 (95% CI = 1.52–5.23, p-value < 0.01) for transitioning from dissatisfied to satisfied with an increased frequency of notification checks. Additionally, the aOR was 3.65 (95% CI = 1.51–8.87, p-value < 0.01) for those expressing interest in a university-provided COVID-19 information database. These findings highlight the importance of accessible and regularly updated information services, similarly shown in previous research demonstrated importance of institutional support, communication effectiveness, and heightened risk perception and awareness. This is particularly important in collectivist cultures, where students tend to prefer institution-driven communication.

The combined-effects analysis (Table 5) indicates significant interaction effects on student satisfaction when factoring in class attendance status. For online-only students, vaccination status showed a positive additive effect (RERI of 3.39, p-value = 0.10), while the frequency of campus COVID-19 notifications exhibited a significant negative interaction on a multiplicative scale (ROR = 0.12, p-value < 0.01). These findings suggest that for online-only students, excessive exposure to infection updates may increase anxiety rather than providing reassurance, emphasizing the need for a balanced institutional communication strategy.

### Implications and recommendations

The findings underscore the need for clear, consistent communication strategies and comprehensive institutional support to enhance student health literacy, thereby increasing perceived safety and trust in the institution. Universities should integrate transparent and frequent health communication policies, ensuring that updates on infection prevention and case trends are disseminated through multiple platforms such as emails, mobile applications, and social media, ensuring accessibility and inclusivity for all students. A data-driven approach should be adopted by establishing an infectious disease information database, which allows institutions to monitor student engagement with safety protocols and adjust policies accordingly. Data analytics can help identify gaps in communication strategies and facilitate evidence-based policy improvements. Institutions could also expand mental health resources, such as counseling services and stress-management programs, to support student well-being and mitigate pandemic-related anxiety. Strengthening these resources fosters a supportive learning environment and improves academic engagement.

Beyond institutional policies, universities should adopt flexible academic policies, such as hybrid learning models, to accommodate students with health concerns or logistical challenges. Faculty and staff should be trained in health communication and crisis management to enhance institutional responsiveness to student concerns. Additionally, community engagement initiatives should be expanded. Universities should collaborate with student organizations, parents, and local health authorities to promote policy awareness and compliance. Hosting forums or webinars can facilitate open discussions on safety measures and enhance transparency in decision-making. Finally, to ensure continuous improvement, institutions should establish evaluation and feedback mechanisms, such as student surveys and institutional reviews, to assess policy effectiveness and student perceptions. This feedback loop enables adaptive policy development to better meet student needs.

### Limitations and future directions

This study has several limitations. First, while degree level (undergraduate or graduate) and faculty affiliation were collected to adjust for demographic variations, individual data on age, gender, race, and nationality were not collected. Although institutional statistics provide a broader demographic context, the absence of specific demographic data limits subgroup analyses. Future research should consider using non-identifiable demographic indicators to examine potential differences in student satisfaction while ensuring anonymity. Additionally, as this study was conducted at a single institution in South Korea, findings may not be fully generalizable to universities with different student compositions or public health policies. Expanding research to multiple institutions across various educational systems would provide broader insights into student satisfaction with infection prevention policies. This study was also cross-sectional, with data collected before the full resumption of in-person classes. As student perceptions may have evolved over time, longitudinal studies are needed to track how satisfaction and engagement with institutional policies change as COVID-19 measures evolve. A long-term observational approach could offer a comprehensive understanding of how policy adjustments influence student trust and compliance with safety measures.

## Conclusion

This study assessed the factors influencing student satisfaction with university guidelines during the expansion of in-person classes as part of a hybrid learning transition amid the COVID-19 pandemic. The findings highlight the critical role of institutional communication and support in shaping student health literacy, perceived safety, and trust in institutional policies. These factors were particularly significant in collectivist cultural settings, where students tend to rely on institution-driven communication rather than independent information-seeking.

Consistent with global research, the results demonstrate that health literacy, reinforced by effective institutional communication and support, is a key determinant of student satisfaction with in-person learning policies. Students who engaged more actively with university-provided information reported greater satisfaction, emphasizing the need for structured, accessible communication strategies in public health crisis management.

By focusing on the transition back to on-campus learning, this study provides practical insights for universities seeking to improve student satisfaction and policy effectiveness in future public health challenges. The findings highlight the importance of data-driven communication strategies, institutional flexibility, and comprehensive student support systems to maintain high-quality education and student well-being during pandemic-related disruptions.

## Electronic supplementary material

Below is the link to the electronic supplementary material.

Supplementary Material 1.

## Data Availability

The data supporting the findings of this study are available within the article and its supplementary materials. Additional data that support the findings of this study are available from the corresponding author upon reasonable request.
