# Supplementary material for: Assessing student satisfaction with university policies related to in-person classes in the era of COVID-19: a case study from Korea
Source: Sci Rep. 2025 Mar 22;15:9876. doi: 10.1038/s41598-025-92360-7 (PMC11928733; doi:10.1038/s41598-025-92360-7)
Supplement: Supplementary file 1 — Supplementary Material 1. [file 41598_2025_92360_MOESM1_ESM.docx]

*Supplementary material for*

Assessing Student Satisfaction with University Policies Related to In-Person Classes in the era of COVID-19: A Case Study from Korea

**Sungyo Jung^1^, Yoojin Cho^1^, Jinhyun Kwon^1^, Yeram Yang^1^, Jaewon Lee^1^, Sungkyoon Kim^1,2^*

^1^Department of Environmental Health Sciences, Graduate School of Public Health, Seoul National University, 1802, Nambusunhwan-ro, Gwanak-gu, Seoul, 08786, Republic of Korea

^2^Institute of Health and Environment, Seoul National University, 1802, Nambusunhwan-ro, Gwanak-gu, Seoul, 08786, Republic of Korea

# **Table captions**

**Table S1.** Survey questionnaire items and corresponding response results.

**Table S2**. Categorization of variables for relative excess risk due to interaction (RERI) and ratios of odds ratios (RORs) analysis: assessing combined effects of in-person class attendance on student satisfaction (N=386).

**Table S3.** Results from multiple logistic regression analysis incorporating in-person class attendance as an independent variable.

**Table S 1.** Survey questionnaire items and corresponding response results.

| Question | Category | Answer | | Response (%) |
| --- | --- | --- | --- | --- |
| Q1 | Degree course | Undergraduate | | 285 (73.83) |
|  |  | Graduate | | 101 (26.17) |
| Q1-1 | Faculty affiliation | College of Humanities | | 50 (12.95) |
|  |  | College of Social Sciences | | 58 (15.03) |
|  |  | College of Natural Sciences | | 26 (6.74) |
|  |  | College of Nursing | | 8 (2.07) |
|  |  | College of Business Administration | | 18 (4.66) |
|  |  | College of Engineering | | 67 (17.36) |
|  |  | College of Agriculture and Life Sciences | | 30 (7.77) |
|  |  | College of Fine Arts | | 3 (0.78) |
|  |  | College of Education | | 43 (11.14) |
|  |  | College of Human Ecology | | 20 (5.18) |
|  |  | College of Veterinary Medicine | | 7 (1.81) |
|  |  | College of Pharmacy | | 4 (1.04) |
|  |  | College of Music | | 6 (1.55) |
|  |  | College of Medicine | | 11 (2.85) |
|  |  | College of Liberal Studies | | 10 (2.59) |
|  |  | School of Law (including Graduate) | | 4 (1.04) |
|  |  | Graduate School of Public Health | | 10 (2.59) |
|  |  | Graduate School of Public Administration | | 2 (0.52) |
|  |  | Graduate School of Environmental Studies | | 1 (0.26) |
|  |  | Graduate School of Convergence Science and Technology | | 1 (0.26) |
|  |  | School of Dentistry (Graduate) | | 7 (1.81) |
| Q2 | Vaccination status | No | | 118 (30.57) |
|  |  | < 14 days after 1^st^ | | 75 (19.43) |
|  |  | > 14 days after 1^st^ | | 63 (16.32) |
|  |  | < 14 days after 2^nd^ | | 73 (18.91) |
|  |  | > 14 days after 2^nd^ | | 57 (14.77) |
| Q3 | Experience of taking  on-campus COVID-19 test | No | | 87 (22.54) |
|  |  | Yes | | 299 (77.46) |
| Q4 | Checking message notifying  information about COVID-19  infection cases on campus | Never | | 35 (9.07) |
|  |  | Seldom | | 136 (35.23) |
|  |  | Often (2-3 times in a week) | | 145 (37.56) |
| Q5 | Information Source  (transition to in-person classes) | News | | 39 (10.10) |
|  |  | Social media | | 62 (16.06) |
|  |  | E-mail (University account) | | 162 (41.97) |
|  |  | School newsletter | | 8 (2.07) |
|  |  | School notice | | 38 (9.84) |
|  |  | School community | | 13 (3.37) |
|  |  | Acquaintance | | 58 (15.03) |
|  |  | Never encountered | | 6 (1.55) |
| Q6 | Perceived information about  university guidelines | Never checked | | 143 (37.05) |
|  |  | Know some | | 215 (55.70) |
|  |  | Know everything | | 28 (7.25) |
| Q6-1 | Information Source  (changed university guidelines) | News | | 15 (6.20) |
|  |  | SNS | | 44 (18.18) |
|  |  | E-mail (University account) | | 55 (22.73) |
|  |  | School newsletter | | 17 (7.02) |
|  |  | School notice | | 84 (34.71) |
|  |  | School community | | 7 (2.89) |
|  |  | Acquaintance | | 20 (8.26) |
| Q7 | ‘In-Person' classes attendance | No | | 121 (31.35) |
|  |  | Yes | | 265 (68.65) |
| Q8 | Satisfaction with overall  guidelines of the university | Very dissatisfied | | 73 (18.91) |
|  |  | Dissatisfied | | 111 (28.76) |
|  |  | Neutral | | 124 (32.12) |
|  |  | Satisfied | | 64 (16.58) |
|  |  | Very satisfied | | 14 (3.63) |
| Q8-1 | Satisfaction with each specific  guidelines of the university | Checking body temperature | Very dissatisfied | 15 (3.89) |
|  |  |  | Dissatisfied | 31 (8.03) |
|  |  |  | Neutral | 75 (19.43) |
|  |  |  | Satisfied | 156 (40.41) |
|  |  |  | Very satisfied | 109 (28.24) |
|  |  | Recording access list | Very dissatisfied | 22 (5.07) |
|  |  |  | Dissatisfied | 35 (9.07) |
|  |  |  | Neutral | 84 (21.76) |
|  |  |  | Satisfied | 142 (36.79) |
|  |  |  | Very satisfied | 103 (26.68) |
|  |  | Wearing a mask | Very dissatisfied | 13 (3.37) |
|  |  |  | Dissatisfied | 19 (4.92) |
|  |  |  | Neutral | 64 (16.58) |
|  |  |  | Satisfied | 119 (30.83) |
|  |  |  | Very satisfied | 171 (44.30) |
|  |  | Periodic ventilation | Very dissatisfied | 148 (38.34) |
|  |  |  | Dissatisfied | 117 (30.31) |
|  |  |  | Neutral | 75 (19.43) |
|  |  |  | Satisfied | 31 (8.03) |
|  |  |  | Very satisfied | 15 (3.89) |
|  |  | Placing hand sanitizer | Very dissatisfied | 10 (2.59) |
|  |  |  | Dissatisfied | 23 (5.96) |
|  |  |  | Neutral | 79 (20.47) |
|  |  |  | Satisfied | 137 (35.49) |
|  |  |  | Very satisfied | 137 (35.49) |
| Q9 | Worried place where COVID-19  is most likely to spread  (multiple choices) | Lecture room, laboratory | | 209 (19.42) |
|  |  | Library | | 114 (10.59) |
|  |  | Dormitory | | 140 (13.01) |
|  |  | Convenience facilities (e.g., restaurants, cafes) | | 344 (31.97) |
|  |  | Indoor and outdoor sports facilities | | 108 (10.04) |
|  |  | Shuttle bus | | 157 (14.59) |
|  |  | Restroom | | 3 (0.28) |
|  |  | None | | 1 (0.09) |
| Q10 | Most worried matters  when using school facilities  about COVID-19 infection  (multiple choices) | Filtration efficiency of masks | | 89 (9.36) |
|  |  | Proper wearing of masks | | 251 (26.39) |
|  |  | Hands sanitizing | | 177 (18.61) |
|  |  | Disinfection of personal items | | 53 (5.57) |
|  |  | Clean and ventilate the surrounding area | | 168 (17.67) |
|  |  | Physical distancing in school facilities | | 211 (22.19) |
|  |  | Others | | 2 (0.21) |
| Q11 | Searching experience for  COVID-19 information | No | | 279 (72.28) |
|  |  | Yes | | 107 (27.72) |
| Q12 | Source of acquiring  COVID-19 information | Korean government agencies | | 61 (28.64) |
|  |  | Overseas organizations (e.g., WHO, CDC) | | 36 (16.90) |
|  |  | Literature searching sites  (e.g., Google scholar, PubMed) | | 47 (22.07) |
|  |  | Media reports or articles | | 68 (31.92) |
|  |  | Others | | 1 (0.47) |
| Q12-1 | Frequency of acquiring  COVID-19 information | Temporary | | 38 (35.51) |
|  |  | < 1 month | | 16 (14.95) |
|  |  | 2-3 times in a week | | 15 (14.02) |
|  |  | 2-3 times in a month | | 38 (35.51) |
| Q13 | The reason you haven’t  search the information  (multiple choices) | Didn’t feel the need to acquire information | | 223 (71.94) |
|  |  | Difficult to use of database | | 76 (24.52) |
|  |  | Others | | 11 (3.55) |
| Q14 | Hoping to use COVID-19  information database  provided by the university. | No | | 104 (26.94) |
|  |  | Yes | | 282 (73.06) |
| Q 15 | Information you want to obtain  by using COVID-19 database  (multiple choices) | Origin of the COVID-19 virus | | 58 (5.36) |
|  |  | Route of infection and transmission  of the COVID-19 virus | | 206 (19.02) |
|  |  | How to prevent COVID-19 infection | | 165 (15.24) |
|  |  | Efficacy and side effects of  the COVID-19 vaccine | | 245 (22.62) |
|  |  | Group immunity, facility capacity and  number of people in accommodation facilities | | 205 (18.93) |
|  |  | Mutated virus of the COVID-19 virus | | 199 (18.37) |
|  |  | Others | | 6 (0.55) |

* The sample size for each questionnaire was 386.

**Table S 2.** Categorization of variables for relative excess risk due to interaction (RERI) and ratios of odds ratios (RORs) analysis: assessing combined effects of in-person class attendance on student satisfaction (N=386).

| Question  number | Category | Answer | Response (%) |
| --- | --- | --- | --- |
| Q1 | Degree course | Undergraduate student | 285 (73.83) |
|  |  | Graduate student | 101 (26.17) |
| Q2 | Vaccination Status | No | 118 (30.57) |
|  |  | Yes (1^st^ or 2^nd^) | 268 (69.43) |
| Q3 | Experience of taking on-campus COVID-19 test | No | 87 (22.54) |
|  |  | Yes | 299 (77.46) |
| Q4 | Checking message notifying information about | Never & Seldom | 171 (44.3) |
|  | COVID-19 infection cases on campus | Often & Everytime | 215 (55.70) |
| Q6 | Perceived information regarding | Never checked | 143 (37.05) |
|  | university guidelines | Know some or everything | 243 (62.95) |
| Q7 | ‘In-Person' classes attendance | No | 121 (31.35) |
|  |  | Yes | 265 (68.65) |
| Q8 | Satisfaction with overall guidelines | Unsatisfied | 73 (18.91) |
|  | of the university | Neutral or Satisfied | 313 (81.09) |
| Q11 | Searching experience for | No | 279 (72.28) |
|  | COVID-19 information | Yes | 107 (27.72) |
| Q14 | Hoping to use COVID-19 information | No | 104 (26.94) |
|  | database provided by the university | Yes | 282 (73.06) |

**Table S 3.** Results from multiple logistic regression analysis incorporating in-person class attendance as an independent variable.

|  | **MODEL I** | | | **MODEL II** | | | **MODEL III** | | | |
| --- | --- | --- | --- | --- | --- | --- | --- | --- | --- | --- |
|  | **OR** | **95% CI** | **P-value** | **aOR^*^** | **95% CI** | **P-value** | **aOR^*^** | **95% CI** | **P-value** |  |
| **‘In-Person' classes attendance** | | | | | | | | | | |
| Unsatisfied | Rf |  |  | Rf |  |  | Rf |  |  |  |
| Neutral | 2.92 | 1.52-5.61 | <0.01** | 2.81 | 1.45-5.44 | <0.01** | 2.83 | 1.43-5.61 | <0.01** |  |
| Satisfied | 1.29 | 0.57-2.88 | 0.54 | 1.18 | 0.52-2.67 | 0.68 | 1.1 | 0.46-2.59 | 0.83 |  |
| **Vaccination status** | | | | | | | | | | |
| Unsatisfied | Rf |  |  | Rf |  |  | Rf |  |  |  |
| Neutral | 1.20 | 0.82-1.75 | 0.36 | 1.18 | 0.80-1.73 | 0.40 | 1.23 | 0.82-1.83 | 0.32 |  |
| Satisfied | 1.78 | 1.11-2.88 | <0.05* | 1.75 | 1.08-2.83 | <0.05* | 1.83 | 1.10-3.03 | <0.05* |  |
| **Experience of taking on-campus COVID-19 test** | | | | | | | | | | |
| Unsatisfied | Rf |  |  | Rf |  |  | Rf |  |  |  |
| Neutral | 1.28 | 0.65-2.51 | 0.47 | 1.29 | 0.66-2.53 | 0.46 | 1.26 | 0.61-2.60 | 0.53 |  |
| Satisfied | 1.93 | 0.89-4.18 | 0.10 | 1.95 | 0.90-4.24 | 0.09 | 1.61 | 0.71-3.67 | 0.25 |  |
| **Checking message notifying information about COVID-19 infection cases on campus** | | | | | | | | | | |
| Unsatisfied | Rf |  |  | Rf |  |  | Rf |  |  |  |
| Neutral | 1.74 | 1.19-2.55 | <0.01** | 1.69 | 1.15-2.48 | <0.01** | 1.44 | 0.94-2.20 | 0.09 |  |
| Satisfied | 2.83 | 1.67-4.77 | <0.01** | 2.71 | 1.59-4.60 | <0.01** | 2.40 | 1.36-4.22 | <0.01** |  |
| **Perceived information regarding university guidelines** | | | | | | | | | | |
| Unsatisfied | Rf |  |  | Rf |  |  | Rf |  |  |  |
| Neutral | 0.73 | 0.47-1.13 | 0.16 | 0.71 | 0.45-1.11 | 0.13 | 0.68 | 0.42-1.09 | 0.11 |  |
| Satisfied | 0.72 | 0.42-1.24 | 0.24 | 0.70 | 0.41-1.20 | 0.20 | 0.53 | 0.29-0.95 | <0.05* |  |
| **Searching experience for COVID-19 information** | | | | | | | | | | |
| Unsatisfied | Rf |  |  | Rf |  |  | Rf |  |  |  |
| Neutral | 0.79 | 0.44-1.43 | 0.44 | 0.77 | 0.43-1.39 | 0.39 | 0.62 | 0.32-1.19 | 0.15 |  |
| Satisfied | 1.47 | 0.74-2.90 | 0.27 | 1.41 | 0.71-2.81 | 0.32 | 1.08 | 0.51-2.31 | 0.84 |  |
| **Hoping to use COVID-19 database provided by the university** | | | | | | | | | | |
| Unsatisfied | Rf |  |  | Rf |  |  | Rf |  |  |  |
| Neutral | 2.85 | 1.65-4.95 | <0.01** | 2.81 | 1.62-4.88 | <0.01** | 3.14 | 1.71-5.78 | <0.01** |  |
| Satisfied | 3.66 | 1.77-7.58 | <0.01** | 3.58 | 1.73-7.43 | <0.01** | 3.10 | 1.40-6.82 | <0.01** |  |
| * Model 1: univariate analysis of each independent variable.  * Model 2: Model 1 adjusted by demographic characteristic  * Model 3: multivariable model incorporating all independent variables. | | | | | | | | | | |

* aOR: adjusted odds ratio; CI: Confidence interval.
